# Separable but Correlated: The Role of Executive Functions and Effortful Control in the Transition to School Age

**DOI:** 10.3390/bs15070845

**Published:** 2025-06-23

**Authors:** Larissa K. Predy, Daphne Vrantsidis, Mahsa Khoei, Naaila Ali, Sandra A. Wiebe

**Affiliations:** Department of Psychology, University of Alberta, Edmonton, AB T6G 2R3, Canada; lpredy@ualberta.ca (L.K.P.);

**Keywords:** early childhood, cohort-sequential longitudinal model, transitions, executive functions, effortful control, internalizing, externalizing, self-regulation, measurement

## Abstract

Executive function (EF) and effortful control (EC) are two similarly defined constructs implicated in self-regulation. Recent debates have questioned whether EF and EC may in fact represent a single construct, and they have undergone scrutiny regarding construct independence. Efforts to differentiate them have further queried whether one may in fact precede the other in early childhood. In a cohort-sequential study of 191 typically developing 4-to-7 year olds (97 girls, 59.7% White), confirmatory factor analysis supported the correlated yet separable two-factor structure of EF and EC with partial scalar invariance across preschool and school-age groups. Longitudinal multi-group modeling was then used to identify predictive pathways between EF, EC, and psychopathology. For both developmental groups, EF predicted externalizing behaviors one year later while EC did not directly predict behavioral outcomes. Internalizing behaviors were found to be highly stable and predictable over time and across age; however, externalizing behaviors significantly predicted internalizing behaviors one year later in the school-age group but not the preschool group. These findings have implications for the measurement of EF and EC in early childhood, as well as the development and prediction of internalizing and externalizing behaviors across the transition to school.

## 1. Introduction

Children’s self-regulation is a topic of high interest to researchers, educators, parents, and clinicians alike. Self-regulation is a complex construct that is generally reflective of an individual’s capacity to manage and adapt their own behavior and emotions and that develops rapidly in early childhood, continuing through early adulthood (Gross, 2015; Montroy et al., 2016). This ability has significant clinical implications over time; in fact, evidence suggests that components of self-regulation often predict later psychopathology (McNeilly et al., 2021; Robson et al., 2020). However, inconsistent operationalized definitions across fields reflect the lack of a cohesive framework of self-regulation and thus variability in the predictions made during this important stage of development. Specifically, when comparing psychological constructs, principles of measurement rely on the assumption that the chosen indicators of separate constructs indeed reflect separate domains of behavior. The specificity of findings diminishes when indicators lack clear differentiation and are instead measuring the same construct labeled differently (i.e., Jangle fallacy; Kelley, 1927).

In recent decades, developmental scientists focused on temperament and neurocognition have recognized a conceptual overlap in their terminology for the mechanisms of self-regulation. From a neurocognitive perspective, executive functioning (EF) is defined as the ability to regulate attention, memory, and prepotent responses in pursuit of goal-directed activities (Diamond, 2013; Espy et al., 2016). From a temperament perspective, effortful control (EC) is defined as the ability to regulate emotional reactivity and impulsive responding (Rothbart et al., 2001). Many researchers have called for better differentiation of EF and EC (e.g., Doebel, 2020; Kim-Spoon et al., 2019; Nigg, 2017). However, measurement methods have varied across studies and, perhaps as a result, findings have been inconsistent as to whether EF and EC are indeed separable or a common construct. While research on middle childhood and later has largely found that EF and EC are separable (e.g., Tiego et al., 2018), findings in early childhood are more mixed (e.g., Dong et al., 2024). In order to clarify our understanding of these self-regulatory systems in early childhood, we must revisit the question of their separability and stability within this unique developmental period.

Major shifts in physical, cognitive, and social functioning occur during the preschool to school-age transition (Bukhalenkova et al., 2022). By school age, children show prominent and identifiable interindividual differences in emotional and behavioral self-regulation. However, disagreement still exists as to the role of underlying cognitive mechanisms of emotional and behavioral self-regulation during the early childhood transition to school age (Barrett et al., 2013). Children’s transition from preschool to school age is rife with change, through which they must adapt to new expectations, rules, people, and physical environments (Tudge et al., 2017). This transition period has been referred to as the 5–7 Year Shift, first labeled as such by the developmental psychologist Sheldon White in 1965 (Sameroff & Haith, 1996). This transition period has been singled out as it introduces many unique shifts in capability and the environmental stressors and challenges associated with increasing expectations for learning and attention. Such shifts have been explored for their influence on children’s emotional, physiological, and stress-related behaviors for decades (Wapner & Kaplan, 2024). While self-regulation develops rapidly during the preschool stage, the expectation of children to regulate their attention, integrate and update rules, and maintain focus on single tasks increases substantially by the first year of school (Chevalier & Clark, 2018; Garon et al., 2008). These shifts signal the importance of the transition from preschool to school age in development and highlight the importance of well-defined constructs and measurement terms for understanding the mechanisms of self-regulation across this period and their role in internalizing and externalizing behavior outcomes.

In particular, developmental psychopathology is interested in the role of EF and EC in the etiology of internalizing and externalizing behaviors. Stronger self-regulation has been largely associated with the better management of or fewer symptoms of externalizing problems, such as aggression or impulsivity, with notable variability related to internalizing symptoms such as anxiety and sadness (for a review of this literature, see Eisenberg et al., 2010). Untangling the environmental and genetic factors associated with these outcomes can help to refine diagnostic criteria, measurement, and early symptom identification, which are crucial for developing effective treatment regimes.

### 1.1. Conceptualizing Executive Functioning in Early Development

Within both neurocognitive and clinical perspectives, EF is considered to be a multidimensional construct. In clinical settings, EF is most often described by its associated functional impairments and has long been considered transdiagnostically relevant (Snyder et al., 2015). The ability to manage time, initiate and complete tasks, follow through on multi-step instructions, exhibit self-control, shift or adapt from one thing to another, and display organizational skills is considered an observable representation of EF in clinical contexts and is generally assessed using questionnaires about daily functioning (Roth et al., 2015). Neurocognitive research, on the other hand, has converged on a definition of EF as a construct consisting of three separable but correlated primary factors: working memory/updating, set shifting, and inhibitory control (i.e., Unity–Diversity Model; Miyake et al., 2000; Diamond, 2013). Although the three factors are unique (diversity), their shared variance can be used to define a higher-order factor (Miyake et al., 2000) or a Common EF factor (unity) in a bifactor model (Friedman & Miyake, 2017). Each factor is typically assessed using direct tasks in laboratory settings.

The factor structure of EF appears to differentiate over time (see Laureys et al., 2022; Lee et al., 2013; Miyake et al., 2000; Snyder et al., 2015; Wiebe et al., 2008; for a review and meta-analysis see Karr et al., 2018). Yet, in early childhood, EF does not appear to fit the mature three-factor structure (Brydges et al., 2014; Laureys et al., 2022; Wiebe et al., 2008). Preschool-aged children tend to display a simplified unitary construct of EF (Brydges et al., 2014; Wiebe et al., 2008, 2011). Further, at this age, children tend to show floor-level performance on set-2 measures, so task batteries tend to include tasks commonly considered to represent working memory and inhibitory control (Wiebe et al., 2008).

### 1.2. Conceptualizing Effortful Control in Early Development

EC has primarily been studied within temperament literature as an important facet of self-regulation (Rothbart & Bates, 2007; Rueda, 2012). Infant temperament reflects a more genetically influenced reactivity (e.g., surgency and negative affect; Rothbart & Bates, 2007). However, with development, EC is increasingly shaped by experience, involving some degree of conscious intention, considering relevant consequences for behavioral activation or inhibition (Kochanska et al., 2000; Rothbart & Bates, 2007).

EC has most often been measured using questionnaire data in both research and practice (e.g., Children’s Behavior Questionnaire (CBQ); Rothbart et al., 2001), but it has also been measured using direct tasks (e.g., Kochanska et al., 1996; Kochanska, 1997; Murray & Kochanska, 2002). However, Kochanska et al. (2002) expressed concern that EC measurement was hindered by diverging conceptualizations, paradigms, test battery selections, and procedures across studies of EC. More recently, others have argued that this lack of clarity and comparability in the measurement of EC has persisted (Bridgett et al., 2015; Eisenberg et al., 2018).

### 1.3. Separability of EF and EC in Early Development

To better characterize self-regulation in early childhood, it would be useful to understand how EF and EC relate to each other, and how much they overlap. Nigg (2017) addresses this semantic overlap and the conflicting terminology used to examine these constructs in the broad domain of self-regulation across studies. Nigg reflects that conceptualizations of EF and EC seem to change depending on when, how, and from what context the terms are studied. For example, he cites several researchers who have found or implied that when EF is examined in the service of another construct (i.e., self-regulation), EF appears measurably and practically the same as that construct. Nigg notes that EF not only comprises top-down strategies for processing information but responds in a goal-directed fashion when automatized regulatory routines are not or cannot be effectively employed. He goes on to differentiate this from EC, by reflecting that EC tends to exclude factors that are focal to ‘high-level EF’, such as rule-governed decision making, reasoning, and planning (for more on ‘high-level EF’ see Diamond, 2013). He proposes that EC supports self-regulation by providing emotionally salient, goal-relevant information to the executive working memory. Nigg concludes that while EC *facilitates* (i.e., consciously supports) self-regulation, EF involves low-level and high-level cognitive processes that *enable* self-regulation to occur (i.e., makes possible). For example, one study found EC to *facilitate* EF training effects in preschoolers, noting significant group differences in EF as a function of EC, and that higher EC improves attention allocation toward EF tasks (Dong et al., 2024). By this logic, for any complex cognition to occur, one must first be able to focus one’s attention and effort on a specific goal-oriented behavior (EF) as well as engage in intentional, conscious regulation (EC). In other words, while EF and EC are related in the activation of complex cognition and self-regulation, they may remain separable constructs, but clearer differentiation remains necessary (Nigg, 2017).

Importantly, measurement models of EF and EC have tended to use either mixed or matched modes of assessment. In a large cross-sectional study, Kim-Spoon et al. (2019) selected both direct and indirect measures to evaluate the relation between EF and EC in an effort to avoid an over- or under-estimation of the magnitude of associations due to measurement method similarities. To assess EF they used three different direct tasks, one of each from the foundational dimensions proposed by Miyake. EC indicators were drawn from widely used questionnaires in both age groups. However, Kim-Spoon and colleagues identified a “trimmed” structure of EC by including only Inhibitory Control and Attentional Control scores, which substantially improved the factor correlations between EF and EC in their modeling. With this, they found significant correlations between EC and all outcomes of childhood adjustment and psychopathology. As a result, the authors addressed a critical recommendation, suggesting that future research employs multimethod approaches to control for issues of method-confounding.

To further complicate comparisons, EC tasks tend to be similar to those used in EF measurement. For example, delay of gratification tasks, Simon Says, box search tasks, and the Head Toes Knees Shoulders (HTKS) task have all been utilized as tasks for both EF and EC across the literature (Nigg, 2017). Many authors have discussed this overlap, often proposing that due to similarities in both the definitions and tasks used to examine these constructs, that EF and EC may in fact be identical constructs that have simply been labeled differently across neurocognitive and temperament research (Allan & Lonigan, 2011). Herein lies the challenge of interdependence faced by social-science researchers; as definitions shape measurement, measures, in turn, shape our understanding of the construct. If the measures are flawed or poorly selected, our understanding of the construct will be flawed as well.

Tiego et al. (2018) addressed this question directly in early adolescence. They indicate a strong empirical overlap between EF and EC when the constructs are assessed through behavioral ratings. In contrast, only 30% of the variance in EC was explained when direct tasks were used to examine working memory capacity and response inhibition. We were not able to find any studies that explicitly and empirically examined this separability of EF and EC in early childhood using confirmatory factor analysis (CFA). There was one study, but it has since been retracted (Retraction: Editor-in-Chief of Wiley Online Library, 2025).

### 1.4. Externalizing and Internalizing Behaviors

EC and EF have long been associated with the development of features of psychopathology, including both externalizing and internalizing problem behaviors (Eisenberg et al., 2010; Robson et al., 2020). While diagnoses are less prevalent in early childhood, the identification of milder features of psychopathology could provide an opportunity for early identification and intervention prior to a need for diagnosis (Kessler et al., 2007).

Internalizing behavior problems are often linked to traits like low mood, social anxiety, or social withdrawal. However, there is significant variability and a wide range of expectations regarding what internalizing behaviors are considered typical during early development. Murray and Kochanska (2002) explored behavioral outcomes longitudinally from 2.5 to 5.5 years and found that both high EC and low EC were associated with poorer internalizing outcomes. They argued that this nonlinear relation suggests that there are “optimal levels” of control, rather than higher levels of control inherently predicting better mental health.

Externalizing behaviors are often associated with behavioral disorders and neurodevelopmental conditions, like attention deficit/hyperactivity disorder (ADHD). In such cases, externalizing behaviors may be identifiable as early as infancy (Wolraich et al., 2011). A meta-analysis of 150 studies (Robson et al., 2020) showed that self-regulation difficulties during preschool (~age 4 years) predict both internalizing and externalizing problems by school age (~age 8 years). Further, preschool self-regulation was negatively associated with externalizing problems in early school age; by early school age (~age 7–8), self-regulation difficulties were associated with more significant externalizing behaviors at age 13 years and into adulthood. They further uncovered that the selected measurement approach (out of 67 different measures of self-regulation across studies) emerged as a significant moderator of the mean effect of self-regulation on psychopathological outcomes. Specifically, they found that objective task-based assessments were a stronger correlate than parent- or teacher-reports, despite being considered less ecologically valid comparatively (Robson et al., 2020).

Such findings emphasize the need for better construct clarity and measurement invariance in this area. A clear understanding of the construct overlap as well as the developmental trajectories of EF and EC across early childhood is particularly important for understanding their relation to psychopathology.

### 1.5. The Present Study

In the present study, we revisit the factor structure of EF and EC in preschool and school-age children, and test EF and EC as predictors of internalizing and externalizing behaviors one year later. Questionnaire data likely best captures emotionally laden daily functioning, while direct tests best represent executive capacity in controlled settings; therefore, we anticipate EF and EC to be distinct constructs when using a multimethod measurement approach. As such, prior to examining the role of EF and EC as predictors of psychopathology, we first employ confirmatory factor analysis (CFA) to examine the factor structures of EF and EC using a multimodal approach, and explore measurement invariance across developmental cohorts.

Research Question 1: What is the relationship between EF and EC across the preschool to school-age transition using a multimethod approach? We hypothesized that EF and EC would reflect distinct but related constructs in preschoolers (age 4–5 years), as well as school-age children (age 6–7 years).

Research Question 2: Do EF and EC predict mental health outcomes (i.e., externalizing and internalizing behaviors) in preschool and school-age children one year later? Based on previous literature, we hypothesized that stronger EF and EC would predict fewer externalizing and internalizing behaviors one year later, though this relationship was expected to differ across developmental groups.

## 2. Materials and Methods

### 2.1. Sample

The sample consisted of 191 typically developing 4- to 7-year-old children during initial recruitment (Wave 1), enrolled between July 2013 and May 2015. At the one-year follow-up (Wave 2), 109 children remained. Missing data at Wave 2 was due to attrition (9.4%) and missing-by-design (28.3%), as children who were 7 years old at Wave 1 aged out of the study by Wave 2. Children were recruited into two developmental groups spanning early to middle childhood. Cohorts were defined by age at Wave 1 (Preschool Cohort: 4.25–5.75 years; School-Age Cohort: 6.25–7.75 years). Table 1 provides child demographics for this sample.

Family demographics were obtained at Wave 1, with median education levels noted as ‘at least some undergraduate education’ for fathers and ‘undergraduate degree’ for mothers. At Wave 1, 91.1% of fathers and 67% of mothers were employed. The majority of the sample (51.3%) reported an annual income above $90,000, making this the most common (mode) income level, while the mean and median household incomes were in the $70,000–79,000 range per annum. Most parents (59.2%) reported that it was ‘not difficult’ to pay their bills. Most parents (89.6%) reported their marital status as ‘married or common-law’.

Child race and/or ethnicity were reported as follows: White (n = 114), South/Southeast Asian (n = 21), Black (n = 12), Indigenous (n = 5), Arab/West Asian (n = 4), Latin American (n = 4), Multiracial (n = 20), or Other (n = 11). The majority of children in the study were born in Canada (88%); all were English-speaking, with 59.2% routinely exposed to additional languages in the home. The majority of children also attended out-of-home care for at least a couple of hours per week (82.7%), and their family size ranged from 1 to 4 children (*M_children_* = 2.36).

Children completed the Brief Intellectual Ability subscales of the Woodcock–Johnson III Tests of Cognitive Abilities (WJ-III; Woodcock et al., 2001; Houghton Mifflin Harcourt (HMH), Rolling Meadows, IL, USA). Intellectual abilities were positively skewed, distributed heavily above the average range (*M_IQ_* = 109; CI = 95–122; range = 76 to 142).

### 2.2. Methods

Children were individually assessed in the laboratory setting at the University of Alberta for approximately 2 to 2.5 h. The longitudinal design included two waves of data collection, approximately 12 months apart. Following administration of the WJ-III, children were administered a large battery of EF tasks at Wave 1. Of this larger EF battery, 7 EF tasks representing the core features of working memory and inhibitory control (see below for task details) were selected here. Shifting was not included in this model, as preschool-age children tend to show floor-level performance on set-shifting measures (Wiebe et al., 2008).

Parents were present for their child’s assessment and completed questionnaires addressing family demographics, developmental history, and EC at Wave 1, as well as the child’s internalizing/externalizing behaviors at both timepoints. Questionnaires were used to measure EC as it manifests in the real world as opposed to measurable skills in a controlled testing environment, and to reduce bias with regard to the overlap with EF in task-based assessments.

Children were provided a snack break midway through each session, and if children were fatigued or distracted, additional breaks were provided as needed. Sessions were digitally video-recorded to check the fidelity of task administration and the reliability of coding. Computer tasks were presented and scored using E-Prime 2.0 software (Psychology Software Tools Inc., Pittsburgh, PA, USA).

### 2.3. Measures

#### 2.3.1. EF Tasks

The EF task battery was developed to include several tasks measuring each component of EF within Miyake’s (Miyake et al., 2000) and Diamond’s (2013) theoretical frameworks. Of these, three tasks were selected to assess working memory, which is the ability to store and manipulate transient information in the short term to guide ongoing or future behavior (Alloway et al., 2004; Baddeley, 2012). Four tasks were selected to assess inhibitory control, which is the ability to actively suppress a prepotent response, interrupt an activated response, delay a response, or avoid interference in a given contact (Catale et al., 2009; Simpson & Riggs, 2007). The internal consistency reliability for each task was estimated using McDonald’s ω, which is considered more robust than Cronbach’s alpha under conditions of variable item loadings and small trial numbers (Kalkbrenner, 2023). The ω coefficients for each measure ranged from 0.64 to 0.89, indicating acceptable to excellent reliability.

*Working Memory.* The Nebraska Barnyard task (Chevalier et al., 2014) is a computerized adaptation of the Hughes et al. (1998) Noisy Book working memory task. Participants listened to a sequence of animal names and then were asked to recall and press corresponding buttons on a touchscreen. Sounds were paired with each animal button pressed to assist with location memory. Once children were familiar with the location of each animal, the coordinating buttons were replaced with blank color-coded boxes followed by one more practice phase. Blocks of up to three trials were then completed at increasing lengths of presented items; when three responses were incorrect within a block, the task was discontinued. Participants indicated answers by selecting one of the two buttons presented on a Planar PT191MU-BK touch screen monitor (Planar Systems, Hillsboro, OR, USA). The summary score was calculated by dividing the number of correct item responses by the total number of items and summing across all administered span lengths. The internal consistency coefficient for Nebraska Barnyard was ω = 0.83 (3–18 trials per participant depending on performance).

The Listening Recall task (adapted from Gathercole & Pickering, 2000) required participants to listen to a short sentence, state whether the sentence was true or false, and then hold the final word of the sentence in memory for later recall. Participants indicated their true or false answer to the experimenter by pointing to one of two Little People figures: a human figure (true) and a puppy figure (false or silly). At the end of each block of sentences, the participant was asked to recall the final word of each sentence. In the first block, participants recalled one sentence at a time, and in subsequent blocks the number of sentences increased incrementally. The criterion to move from one block to another was >50% correct for the block (three of the four trials); if this criterion was not reached, the task was terminated. This task was manually scored by the experimenter, with a mean inter-rater reliability of 99.24% (based on 18% of assessments). The summary score was calculated by dividing the number of correct item responses by the total number of items. The internal consistency coefficient for Listening Recall was ω = 0.89 (trials ranged from 4–16).

The Word Span task (adapted from Nutley et al., 2009) involved both forward and backward span phases, but only backward span performance was used in this analysis. In the backwards span phase, Tookie the Toucan, a hand puppet, read aloud a sequence of age-appropriate, monosyllabic nouns and participants were asked to recite them in reverse order (e.g., soap-book = book-soap). Each block included three trials starting with a 2-word series, increasing by an increment of one word per block. The participant needed at least one trial correct in a block to move on to the next block; if all three trials were incorrect the task was terminated. If the first two trials were correct, then the third trial was skipped to increase the efficiency of administration and prevent participant fatigue. The summary score was calculated by dividing the number of correct item responses on backward word span items by the total number of backward word span items. The internal consistency coefficient for Backward Word Span was ω = 0.67 (3–12 trials per participant).

*Inhibitory Control.* The Flanker task (adapted from Rueda et al., 2004) presented a child-friendly stimuli with fish as the target and congruent and incongruent flankers, and starfish as the neutral flanker. Congruent trials required participants to “help Fishy and her friends get to school” by focusing on which way Fishy was swimming and ignoring which way her friends were going. Responses were indicated by the participant pressing the left or right button on a Cedrus^®^ RB-530 response pad (Cedrus Corporation, San Pedro, CA, USA). Feedback was provided in the form of bubble sounds for correct responses and a short tone for incorrect or non-response. An initial practice trial allowed the participant to become familiar with the congruent, incongruent, and neutral stimuli, as well as the different forms of feedback, and was followed by a test trial. The selected dependent variable was the accuracy score, calculated by dividing the number of correct trials by the total trials for incongruent trials. The internal consistency coefficient for the Flanker task (incongruent trials) was ω = 0.84 (12 incongruent trials).

The Simon task (adapted from Shing et al., 2010) is a computerized task in which participants are asked to respond to the stimuli presented to the left or right of the computer screen by pressing the left or right button on the button box. The correct response was dictated by the identity of the stimuli. The participant must overcome the prepotent tendency to respond to incorrect information. In an age-appropriate adaptation of the classic Simon task, the “beach sorting game,” participants were asked to help two characters clean up the beach by sorting the beach balls and seashells into piles. The selected dependent variable was the accuracy score calculated by dividing the number of correct trials by the total trials for incongruent trials. The internal consistency coefficient for the Simon task (incongruent trials) was ω = 0.77 (20 incongruent trials).

The Fish Go/No-Go task (Wiebe et al., 2012) is a computerized task that measures the ability to suppress a prepotent response. Participants used a button box to respond to a “go” fish stimuli (75% of trials) and inhibit responses to “no-go” shark stimuli (25% of trials). A limited response time window (1500 ms) maximized inhibitory demand. The selected dependent variable was the d prime (d’) score, which consisted of the standardized difference between hit rates and false alarm rates, calculated by subtracting the z-score value of the hit rate from the z-score value of the false alarm rate. The internal consistency coefficient for the Go/No-Go task was ω = 0.84 (40 trials: 30 Go, 10 No-Go).

The Global–Local task (adapted from Bialystok, 2010) is a computerized task designed to measure attention shifting abilities for the global or local features of a complex stimuli (e.g., a heart made up of small hearts). The task includes inhibitory demands, with children being required to inhibit responses to incongruent stimuli when global and local features are conflicting. Incongruent stimuli are predicted to elicit the greatest inhibitory response because participants must suppress their attention to conflicting stimuli in order to provide a correct response on the touch screen monitor (i.e., ignoring the global attribute when responding to the local attribute and vice versa). The selected dependent variable was the accuracy score, calculated by dividing the number of correct trials by the total trials for incongruent trials. The internal consistency coefficient for the Global–Local task (incongruent trials) was ω = 0.64 (8 incongruent trials).

#### 2.3.2. Effortful Control

The Child Behavior Questionnaire, Short Form (Putnam & Rothbart, 2006), is a questionnaire designed to quantify temperament and self-regulatory traits, and consists of items rated on a 7-point Likert scale ranging from 1 = extremely untrue -to- 7 = extremely true; a not applicable option is also provided. The EC construct was defined by the following subscales: Inhibition (INH = 6-items; α = 0.60), which included such statements as “*can lower his/her voice when asked to do so*”; Attention–Focus (AttF = 6-items; α = 0.73), which included such statements as “*when picking up toys or other jobs, usually keeps at the task until it’s done*”; Low-Intensity Pleasure (LIPL = 8-items; α = 0.68), which included such statements as “*enjoys just being talked to*”; and Perceptual Sensitivity (PERC = 6-items; α = 0.69), which included such statements as “*is quickly aware of some new item in the living room*”. Higher scores on each subscale represent greater self-regulation.

#### 2.3.3. Internalizing and Externalizing Behaviors

The Strengths and Difficulties Questionnaire (SDQ; Muris et al., 2003) is a brief emotional and behavioral screening questionnaire for children and young people that consists of five subscales of items rated on a 3-point Likert-type scale ranging from 1 = Not True -to- 3 = Certainly True. Goodman et al. (2010) suggest that use of the five separate subscales may not be justified in low-risk samples. As such, given the nature of this study’s non-clinical community sample and the proposed research questions, two amalgamated scales were examined from the SDQ to represent indicators of psychopathology: internalizing behaviors (sum of 10 items from emotional symptoms and peer problems subscales; α = 0.59), which includes such statements as “*often unhappy, downhearted*” (emotional) and “*rather solitary, tends to play alone*” (peer problems), and externalizing behaviors (sum of 10 items from the hyperactivity and conduct behavior subscales; α = 0.79), which includes such statements as “*constantly fidgeting or squirming*” (hyperactivity) and “*often has temper tantrums or hot tempers*” (conduct). Higher scores on each subscale represent more difficulty.

#### 2.3.4. Statistical Methods

Descriptive statistics were calculated using SPSS version 29.0.1.0; and confirmatory factor analysis, measurement invariance testing, and longitudinal panel models were conducted in MPlus version 8.4 (Muthén & Muthén; Los Angeles, CA, USA) (Muthén & Muthén, 1998–2017).

*Confirmatory Factor Analysis (CFA).* CFA is a statistical procedure designed to identify the degree to which several factors form a construct. Theorized models are compared to a one-factor model to evaluate model fit (Kline, 2011). In this study, we compared fit indices for specified models of EF and EC to determine the best empirical fit to the data and to determine whether EF and EC would emerge as separable constructs. Model fit criteria were based on the close-fit-hypotheses reported by Little (2013): *χ*^2^ (acceptable fit > 0.05), CFI/TLI (close fit = 0.95–0.99, acceptable fit = 0.90–0.95, mediocre fit = 0.85–0.89), SRMR and RMSEA (close fit = 0.01–0.05, acceptable fit = 0.05–0.08, mediocre fit = 0.08–0.10).

*Measurement Invariance.* Measurement invariance testing is used to determine whether a measurement model is equivalent across specified conditions (e.g., age groups). Models are tested with increasing restrictions, with each successive model retaining the equality constraints of the preceding model. Across conditions, configural invariance examines the equality of the structural model, metric invariance examines the equality of the factor loadings, and scalar invariance examines the equality of the intercepts. In this case, measurement invariance testing was conducted to determine whether EF and EC are stable constructs across developmental groups (i.e., preschool and school age).

*Simple Longitudinal Regression Modelling.* Following confirmation of the baseline measurement model, to fit the conceptual model assessing the associations between EF and EC and changes in the internalizing and externalizing outcomes, we employed a simple longitudinal regression model, with internalizing and externalizing behaviors measured at both Wave 1 and Wave 2 serving as dependent variables. The factor structure (aka the final measurement model) of EF/EC was the independent variable. Following a pruning process to eliminate longitudinally non-significant paths from the model, we then explored whether the remaining predictive paths were moderated by developmental group (i.e., preschool vs. school age).

## 3. Results

### 3.1. Preliminary Analyses

Simple scatter plots were produced and relations among variables and data points were examined for outliers and linearity using the intraocular method (Johnson et al., 2016). The correlations between all indicators and scales considered in the conceptual model are shown in Table 2.

All within-construct correlations were statistically significant. Few statistically significant correlations were present between indicators of EF (tasks) and EC (CBQ subscales): Listening Recall and Nebraska Barnyard (EF) showed small to medium correlations with Attention–Focus (EC). Internalizing and externalizing behaviors each showed statistically significant stability across waves, but only correlated with each other within waves. Several EF indicators showed small negative correlations with externalizing at Wave 1, but only one EF indicator (Go-No-Go) was correlated with externalizing at Wave 2. In contrast, only one EC indicator (Low Intensity Pleasure) was uncorrelated to externalizing across Waves, though correlations were small-to-moderate. EC and EF indicators were not correlated with internalizing at either Wave.

### 3.2. Measurement Model Results

#### 3.2.1. Establishing the Factor Structure of EF and EC

**Figure 1:**
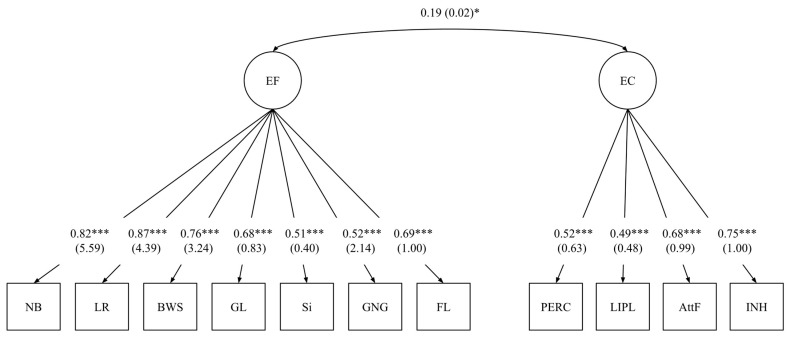
Model 2: Best-fitting model of EF with EC. Standardized (unstandardized) loadings. Note. EF = Executive Functions; EC1 = Effortful Control; NB = Nebraska Barnyard; LR = Listening Recall; BWS = Backward Word Span; GL = Global Local; Si = Simon; GNG = Go No-Go; FL = Flanker; PERC = Perceptual Sensitivity; AttF = Attention–Focus; INH = Inhibitory Control; LIPL = Low-Intensity Pleasure. *** *p* < 0.001, * *p* < 0.05.

To determine the best-fitting model for EF and EC, we tested three models. Model 1 was a unitary factor comprising 11 indicators from direct tasks and rating-scale data. Model 2 was a two-factor model comprising seven direct tasks as indicators of EF and four rating scale indicators of EC. Model 3 was a three-factor model comprising three task-based indicators of working memory, four task-based indicators of inhibitory control, and four rating scale indicators of EC. For Models 2 and 3, all latent factors were allowed to covary. The two-factor model (Figure 1) was identified as the best-fitting model. The model fit indices and model comparisons are shown in Table 3.

#### 3.2.2. Testing the EF/EC Measurement Model Across Developmental Groups

As shown in Table 4, invariance testing supported full configural and metric invariance, and partial scalar invariance. The intercepts for the *Low-Intensity Pleasure* subscale differed between developmental groups. To achieve adequate model fit, the residual for *Low-Intensity Pleasure* was allowed to covary.

### 3.3. Longitudinal Regression Model Results

#### 3.3.1. Predicting Psychopathology with a Longitudinal Regression Model

The EF/EC measurement model, in which EF and EC were allowed to covary, was the basis for the longitudinal regression model with the addition of internalizing and externalizing behavior ratings at Wave 1 and Wave 2. The internalizing and externalizing variables within each wave were allowed to covary, and Wave 2 outcomes were regressed on Wave 1 predictors. The initial model included all possible Wave 1→Wave 2 regression paths. This model showed marginal fit to the data (*χ*^2^ = 229.88, *df* = 175, *p* < 0.003, RMSEA = 0.057 (0.034, 0.077), CFI = 0.90, TLI = 0.89, SRMR = 0.10); however, the examination of modification indices did not reveal any changes that were theoretically justifiable.

Next, we progressively removed paths that were non-significant across both the preschool and school-age groups, testing whether the model fit significantly worsened at each step. This yielded a simplified regression model that was statistically comparable to the original model. Specifically, this eliminated paths from EC and EF→Wave 2 internalizing behaviors, and from EC and Wave 1 internalizing→Wave 2 externalizing behaviors. Paths from EF→Wave 2 externalizing and Wave 1 externalizing→Wave 2 internalizing remained.

#### 3.3.2. Testing Developmental Group as a Moderator

**Figure 2:**
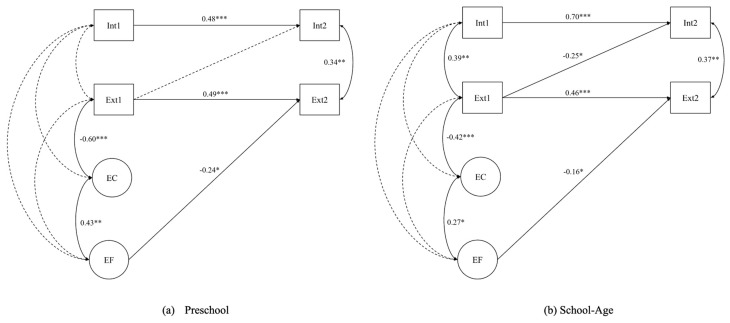
Final longitudinal regression by developmental age. Note. (**a**) Final model for preschool age group (ages 4–5 years); (**b**) Final model for school-age group (ages 6–7 years). Note. Regression paths and correlations are standardized. EF = Executive Function; EC = Effortful Control; Int = Internalizing Behaviors; Ext = Externalizing Behaviors. Wave 1 and 2 are indicated by numerals per variable. *** *p* < 0.001 ** *p* < 0.01 * *p* < 0.05.

To determine whether the final regression paths were moderated by developmental group (preschool vs. school age), each path was systematically constrained to be equal. Constraining the path from EF→Wave 2 externalizing behaviors to be equal did not statistically worsen the model fit (Δ*χ*^2^ = 2.748, Δ*df* = 1, *p* = 0.097). The relationship between these variables does not differ significantly across developmental groups, meaning that age group does not moderate the effect of EF on externalizing behavior one year later. On the other hand, the path from Wave 1 externalizing behaviors→Wave 2 internalizing behaviors could not be constrained to equality (Δ*χ*^2^ = 5.212, Δ*df* = 1, *p* = 0.022). This result indicates that there is a significant moderating effect based on age; specifically, when taking into account earlier internalizing behaviors, externalizing predicts internalizing one year later but only in the school-age group (*p* = 0.032), while there was no significant relationship in the preschool group (*p* = 0.259). Figure 2 shows the final models for both the preschool and school-age groups.

The standardized and unstandardized results of the final regression model are provided in Table 5 for both preschool and school-age groups.

## 4. Discussion

This study examined whether EF and EC are distinct constructs in early childhood and tested whether EF and EC predict behavior problems across the preschool to school-age transition. Confirmatory factor analysis supported a two-factor model in which EF (measured by direct tasks) and EC (measured by parent-report questionnaires) represent separate but related constructs. This two-factor structure held for both preschool and school-age children. Interestingly, EC did not emerge as a significant predictor of psychopathology in the current model in either group, even when controlling for the potential moderating role of EF; however, a small but statistically significant negative relationship was observed between EF and externalizing behaviors one year later when EF and EC were correlated within the model and when accounting for baseline externalizing behaviors. EF consistently emerged as a significant predictor of externalizing behaviors across this developmental period. Another key finding was the significant moderating effect of age on the relationship between externalizing and internalizing behaviors. In school-age children, Wave 1 externalizing behaviors significantly predicted internalizing behaviors one year later; however, this same relationship was not observed in the preschool group.

The school-age transition is a time of profound change in early childhood, particularly in terms of self-regulatory demands. While the importance of self-regulation in early development has been well documented, without confidence in measurement models our understanding of these psychological concepts remains weak (Karr et al., 2018). Concerns over diverging conceptualizations, test battery selection, and procedures across studies of EF and EC have resulted in debate over construct separability. In response, we aimed to contribute to the search for clarity regarding the relationship between EF and EC, and determine how well each predicts internalizing and externalizing behaviors across this early developmental transition when examined using a multimethod CFA approach.

### 4.1. Separability of EF and EC

In the present study, we employed a multimodal measurement approach prior to progressively testing a longitudinal regression model to address the limitations of previous studies (e.g., Kim-Spoon et al., 2019). By standing on several researchers’ proverbial shoulders, we were able to examine this question of construct purity by employing both a mixed method approach and by identifying the factor analytic structure of EF and EC. This comprehensive approach is a strength of the present design that allowed us to delineate complex questions in the literature concerning the early development of these two important and overlapping constructs. Direct performance tasks were also utilized to represent latent EF based on the most commonly employed methodologies in the literature (Nigg, 2017). In contrast, parent questionnaires were utilized to represent theoretical EC based on the assertion that EC may facilitate and represent an emotion-laden experientially tied determinant of self-regulation (Nigg, 2017), thus emphasizing the importance of parental observations of the daily deployment of this domain of cognitive control. Utilizing two different measurement methods (direct tests for EF vs. parent ratings for EC) and theory-driven indicators and factor structures for each proposed definition, we were able to capture the subtle differences proposed for each theoretical construct in this early developmental stage. While our approach mirrored the multimethod approach suggested by Kim-Spoon et al. (2019), it is important to note that this approach also introduced a difference in method variance between the two constructs and examined the full EC factor structure.

We examined unitary versus multi-factor structures for preschool and school-age children. This approach also allowed us to align with proposed standards for modeling EF in early childhood (Karr et al., 2018; Wiebe et al., 2008, 2011) by representing theorized aspects of EF with seven age-appropriate direct tasks related to working memory and inhibitory control. EF and EC emerged as separate but related constructs, revealing a small but significant correlation between EF and EC. In short, our findings confirm that EF and EC are distinct constructs in both preschool and school-age children, which supports the hypothesis that these two constructs, while related, represent separate latent processes.

While our initial findings regarding the separability of EF and EC contradict the proposal that EF may be measurably and practically the same as EC (Nigg, 2017), when EC and EF are correlated within a regression model as represented here, important differences in predictive outcomes emerge, supporting the differential construct validity between preschool and school-age children (the 5–7 year shift). Interestingly, while the relationship between EF and EC was relatively small and showed non-invariance across developmental groups in the full measurement model, within the longitudinal regression model, the relationship between EF and EC was stronger in preschool (B = 0.433, *p* < 0.001) than school-age (B = 0.191, *p* = 0.056) children, suggesting that the relationship may be more important when considering internalizing and externalizing outcomes during the younger preschool transition to school age.

### 4.2. Clinical Relevance of EF and EC in Relation to Psychopathology

Our results revealed that EF and externalizing at Wave 1, but not EC, predicted externalizing behaviors at Wave 2. These results suggest that lab-based tasks are more informative with regard to later externalizing behaviors (e.g., behavior dysregulation, conduct problems) than parent-ratings of EC. It is notable that EC showed a larger negative correlation with externalizing behaviors at the preschool age, and the size of the relationship was lower in the school-age group. It is possible that parental observations of EC in preschoolers more closely resemble behavior dysregulation and conduct problems, as measured by the externalizing scale in this early stage of development.

In contrast, internalizing behaviors were not well predicted by EF or EC in our model. Neither EF nor EC directly predicted internalizing behaviors one year later. However, internalizing behaviors showed significant stability over time, with quite a large correlation coefficient for the school-age group, suggesting that the early identification of and interventions for emotional and peer problems are crucial even at this young age. McNeilly et al. (2021) reported mood dysregulation in childhood (as early as age 6 years) as less predictive of later complex mood problems compared to predictions made in middle schoolers. However, in contrast to the conclusions of McNeilly et al. (2021), internalizing outcomes were in fact predicted well by internalizing ratings one year earlier for both developmental groups within our model. This finding has clinical implications for the reliability of parent-reported internalizing challenges as early as preschool into school age. Further, in the school-age group, externalizing problems were negatively associated with internalizing one year later, suggesting that children with higher internalizing behaviors may show far fewer externalizing behaviors and could be less noticeable. Alternatively, this difference across groups may reflect a tendency to further suppress externalizing behaviors, perhaps over-regulating through the expression of withdrawal.

### 4.3. Theoretical, Methodological, and Practical Implications

Modeling and predicting the outcomes associated with EF in early childhood without the inclusion of a complete model of EC, as measured by parental ratings, may overlook important emotionally laden self-regulatory indicators only obvious in the day-to-day environment. The major difference between EF and EC defined in a “trimmed” manner involving only attention and inhibitory control indicators, as in Kim-Spoon et al. (2019), is perhaps the focus on “cool” aspects of self-regulation versus both the “cool” and “hot” components applicable in vivo. Zelazo et al. (2005) reviewed models of “hot” vs. “cool” EF, which have been studied in depth over the years, indicating that “cool” tasks lack affective or motivational components, whereas “hot” EF is thought to require some degree of emotional processing within the task itself. However, the conceptual link between “hot” EF and the construct of EC specifically has been relatively minimal. Generally, research emphasizing the “hot” and “cool” nature of EF or EC discuss these as inherent to each and tied to specific tasks. To this effect, Lin et al. (2019) proposed a single self-regulatory model using performance-based tasks, from which they suggest that EC tasks may be inherently “hot” while EF tasks may be inherently “cool.” The present study found that the relation between EF and EC contributes to a powerful predictive model of behavioral outcomes across early development. Whether “hot” or “cool”, our findings suggest that both aspects are critical to understanding the trajectory of psychopathology across a preschool to school-age sample. Our study supports that modeling and predictions made from EF should include comprehensive rating-based evaluations of EC, so as not to overlook the important self-regulatory indicators in daily functioning that may contribute to a broader picture of well-being and mental health over time. Both factors appear to be important when considering behavioral outcomes across the transition to school age.

The analytical approach taken in the present study offers a comprehensive means for examining EF and EC across the early developmental period. We posit that findings based on matching measurement modalities of EF and EC (e.g., direct testing only or indirect ratings only) might result in error and lack sensitivity to differentiate the inherent environmental influences reflected in daily performance. Specifically, we suggest that parent ratings of EC (as measured by the CBQ) reflect the observable daily functioning of effortful self-regulatory mechanisms as opposed to the measurable capacity otherwise demonstrated within a controlled testing environment. As such, it is possible that measurement utilizing both questionnaire data and direct testing may be important to capture the nuanced differences between EF and EC across environments. Consistent with this suggestion, preliminary CFA modeling of each factor independently resulted in strong measurement models that were invariant across waves. These findings have important implications for support planning and interventions. Parent ratings of their child’s internalizing behavior as early as age 4 years are stable estimates of later internalizing outcomes, as are externalizing ratings. Parents, teachers, and clinicians may find it prudent to assume internalizing problems based on more observable externalizing behaviors by age 5 years and up, as well as to intervene in relational challenges and emotional dysregulation as early as possible when such behaviors are specifically identified as young as preschool age.

These findings provide a framework for predicting the emergence of both internalizing and externalizing behaviors based on EF abilities and parental endorsements of EC across early development. Such mapping provides insight into the imperative need to teach and support self-regulatory skills early in elementary, by way of expressing and understanding, as opposed to simply masking or suppressing emotions and behavioral expression. Teachers and parents may be able to substantially improve psychopathological outcomes across the preschool to school-age transition by focusing on developing children’s competencies related to social skills, emotional regulation, and cognitive control. Even the variance in academic outcomes for middle school children has been shown to be predicted uniquely by behavioral regulation in the first year of school (Howard et al., 2021). As such, general educational curriculums in preschool through early elementary could be used to incorporate these developmental goals more prominently to improve outcomes across psychopathology and academic achievement.

### 4.4. Limitations and Future Direction

Our study found that preschool and school-age children are mostly a homogenous group in terms of EF and EC despite the various expectations and experiential changes that occur during the 5–7 year shift. However, we did not explore or control for these potential environmental influences on parental ratings of EC or on EF. Future research may consider the inclusion of relevant environmental factors, such as external caregiver experiences, parenting beliefs and attitudes, parental warmth and engagement, as well as child-rearing practices (e.g., screen time, external childcare/socialization).

While a strength of the current study is in the comprehensive CFA approach, using the latent variable model to examine regression paths in a cohort-sequential design, it may still be valuable to examine EF and EC using a 4 × 4 design with comparable psychometrics for both constructs to improve the replicability of findings and test for all possible influences. Unfortunately, when EC has been assessed using laboratory tasks, the tasks themselves have been either identical or extremely similar to EF tasks. Therefore, method overlap becomes a major concern. Our use of task-based and questionnaire modalities allowed us to explore parental endorsements of real-life EC in young children and observe how such skills are reflected in daily living. However, perhaps task development related to the emotional endorsements and peer problems associated with EC would be possible and future research may well examine EF and EC experimentally by addressing these same features without the risk of falling victim to the Jangle fallacy (Kelley, 1927). Examining differences across this transition period with both direct and indirect measures of EF and EC (i.e., EF questionnaire, EC questionnaire, EF tasks, EC tasks) would allow for a comprehensive in-depth analysis comparing the factor structure of a combined model of EF and EC at various stages of early development.

Finally, there is a clear need for further research to clarify the interplay between EF, EC, and psychopathology across early development. In particular, understanding how the relationship between EF and EC changes across a higher frequency or additional waves of data may provide insights into the dynamic interplay between the constructs throughout early development.

## 5. Conclusions

This study and its findings provide clear evidence of the relationship between EF and EC and underscores the distinct nature of EF and EC in predicting children’s emotional and behavioral development. It highlights the significant role of EF in mitigating externalizing behaviors and recognizes the importance of early identification and measurement, including the complex interplay between factors related to self-regulation. Utilizing this approach, continued research in this area is crucial to inform the development of targeted interventions that bolster all aspects of children’s self-regulation to support their healthy emotional and behavioral development.

## Figures and Tables

**Table 1:** Child demographics.

	Wave 1				Wave 2			
Cohort	N	% Female	Age Range	*M_age_*	N	% Female	Age Range	*M_age_*
Sample	191	50.8%	4.25–7.75	6.01	109	54.1%	5.25–7.75	5.47
Preschool	83	51.8%	4.25–5.75	4.85	66	53%	5.25–6.75	4.88
School Age	108	50%	6.25–7.75	6.90	43	55.8%	7.25–7.75	6.38

**Table 2:** Descriptive statistics and correlations across waves.

	M	SD	N	Fl	GL	GNG	Si	LR	NB	BWS	AttF	INH	LIPL	PERC	Ext1	Int1	Ext2	Int2
Fl	0.814	0.222	182	1														
GL	0.726	0.190	186	0.422 **	-													
GNG	3.180	0.631	179	0.433 **	0.365 **	-												
Si	0.868	0.122	180	0.272 **	0.387 **	0.356 **	-											
LR	1.400	0.758	177	0.565 **	0.591 **	0.399 **	0.453 **	-										
NB	3.119	1.048	184	0.532 **	0.486 **	0.393 **	0.356 **	0.697 **	-									
BWS	1.222	0.647	179	0.479 **	0.493 **	0.301 **	0.322 **	0.603 **	0.635 **	-								
AttF	4.935	1.033	191	0.141	0.106	0.127	0.132	0.244 **	0.283 **	0.137	-							
INH	5.029	0.940	191	0.063	0.079	0.100	0.041	0.094	0.132	0.020	0.524 **	-						
LIPL	5.904	0.684	191	−0.098	−0.123	−0.057	−0.087	−0.025	0.023	−0.085	0.336 **	0.334 **	-					
PERC	5.560	0.853	191	0.067	−0.023	0.038	0.085	0.011	0.069	−0.01	0.278 **	0.397 **	0.375 **	-				
Ext1	5.330	3.547	191	−0.159 *	−0.187 *	−0.163 *	−0.144	−0.152 *	−0.163 *	−0.147 *	−0.406 **	−0.393 **	−0.045	−0.153 *	-			
Int1	2.974	2.416	191	−0.006	0.003	0.000	0.009	−0.081	−0.058	−0.080	−0.091	−0.103	−0.001	−0.010	0.304 **	-		
Ext2	5.083	3.473	108	−0.078	−0.096	−0.260 **	−0.184	−0.119	−0.169	−0.039	−0.352 **	−0.418 **	−0.124	−0.225 *	0.526 **	0.187	-	
Int2	2.925	2.586	108	0.091	0.034	−0.186	0.128	0.094	0.051	0.144	−0.067	0.015	0.114	0.062	0.170	0.551 **	0.314 **	-

Note: Fl = Flanker; GL = Global Local; GNG = Go No-Go; Si = Simon; LR = Listening Recall; NB = Nebraska Barnyard; BWS = Backward Word Span; AttF = Attention–Focus; INH = Inhibitory Control; LIPL = Low-Intensity Pleasure; PERC = Perceptual Sensitivity; Int = Internalizing Behaviors; Ext = Externalizing Behaviors; Wave 1 and 2 are indicated by numeral per variable. ** *p* < 0.01, * *p* < 0.05.

**Table 3:** Confirmatory factor analysis of EF and EC at Wave 1.

	Model Fit Indices							Model Comparisons			
MODEL	*χ* ^2^	*df*	*p*	RMSEA	CFI	TLI	SRMR	Models	Δ*χ*^2^	*df*	*p*
Model 1: 1-Factor EF+EC	180.00	44	<0.001	0.127 (0.108, 0.147)	0.79	0.73	0.11	-	-	-	-
Model 2: 2-Factor EF/EC	59.59	43	0.048	0.045 (0.005, 0.071)	0.97	0.97	0.06	1 vs. 2	120.40	1	<0.001
Model 3: 3-Factor WM/IC/EC	56.67	41	0.053	0.045 (0.000, 0.071)	0.98	0.97	0.06	2 vs. 3	2.92	2	0.232

Note: EF = Executive Functions; EC = Effortful Control; WM = Working Memory; IC = Inhibitory Control.

**Table 4:** Invariance testing across developmental groups for the EF/EC measurement model.

	Model Fit Indices							Model Comparisons			
MODEL	*χ* ^2^	*df*	*p*	RMSEA	CFI	TLI	SRMR	Δ*χ*^2^	*df*	*p*	Accepted?
Configural Invariance	107.58	86	0.058	0.051 (0.000, 0.080)	0.95	0.93	0.07	-	-	-	-
Metric Invariance	124.57	95	0.023	0.057 (0.023, 0.083)	0.93	0.92	0.10	16.99	9	0.049	Yes
Scalar Invariance	143.98	104	0.006	0.063 (0.035, 0.087)	0.90	0.90	0.11	19.41	9	0.022	No
Partial Scalar Invariance	133.92	103	0.022	0.056 (0.023, 0.081)	0.93	0.92	0.11	9.34	8	0.314	Yes

**Table 5:** Final longitudinal regression model: standardized and unstandardized results.

	Preschool				School Age			
MODEL	B	SE	*p*	*β*	B	SE	*p*	*β*
EF WITH EC	0.433	0.126	0.001	0.433 **	0.191	0.100	0.056	0.272 **
Int2 on Int1	0.521	0.106	<0.001	0.484 ***	0.720	0.124	<0.001	0.701 ***
Ext2 on Ext1	0.503	0.106	<0.001	0.492 **	0.418	0.104	<0.001	0.455 ***
Int2 on Ext1	−0.092	0.082	0.264	0.125	−0.177	0.080	0.026	−0.251 *
Ext2 on EF	−0.860	0.352	0.015	−0.237 *	−0.860	0.352	0.015	−0.162 *
Ext1 WITH EF	−0.770	0.417	0.065	−0.217	−0.414	0.245	0.091	−0.196
Ext1 WITH EC	−2.116	0.415	<0.001	−0.596 ***	−1.717	0.513	0.001	−0.424 ***
Int1 WITH Ext1	1.681	0.960	0.080	0.196	3.287	0.867	<0.001	0.392 ***
Int1 WITH EC	−0.406	0.313	0.194	−0.168	−0.231	0.307	0.452	−0.083
Int1 WITH EF	−0.537	0.284	0.058	−0.222	−0.006	0.165	0.969	0.004
Int2 WITH Ext2	2.206	0.862	0.010	0.339 **	1.904	0.853	0.026	0.368 **

Note. Wave 1 and 2 are indicated by numerals per variable. EF = Executive Function; EC = Effortful Control; Int = Internalizing Behaviors; Ext = Externalizing Behaviors; LIPL = Low-Intensity Pleasure; PERC = Perceptual Sensitivity. *** *p* < 0.001 ** *p* < 0.01 * *p* < 0.05.

## Data Availability

Data from this study cannot be publicly shared because of ethical restrictions.

## Abbreviations

The following abbreviations are used in this manuscript:

CFA	Confirmatory Factor Analysis
EC	Effortful Control
EF	Executive Function
RMSEA	Root Mean Square Error of Approximation
CFI	Comparative Fit Index
TLI	Tucker Lewis Index
SRMR	Standardized Root Mean Square Residual
